# The Importance of Echocardiographic Persistence in Predicting Apical Aneurysms via Color Doppler in Hypertrophic Cardiomyopathy

**DOI:** 10.7759/cureus.107801

**Published:** 2026-04-27

**Authors:** Muhammed Emre Gülesir, Berkay Topal, Umut Ata Ugras, Ertugrul Aribas

**Affiliations:** 1 Cardiology, Bolu Abant Izzet Baysal University, Bolu, TUR; 2 Cardiovascular Surgery, Bolu Abant Izzet Baysal University, Bolu, TUR

**Keywords:** apical hypertrophic cardiomyopathy, color doppler, left ventricular apical aneurysm, myocardial bridge, sudden cardiac death, transthoracic echocardiography

## Abstract

In clinical practice, apical hypertrophic cardiomyopathy (HCM) is an insidious phenotype that often conceals significant structural complications. Our case demonstrates a critical "clinical pearl": the necessity of echocardiographic persistence. While standard apical four-chamber views failed to reveal a large apical aneurysm, our clinical suspicion - triggered solely by a high-velocity apical color Doppler jet - led to meticulous probe manipulations (intercostal "sliding" and "tilt") that eventually unmasked a characteristic "hourglass" aneurysmal sac. Beyond the diagnostic challenge, this case highlights a significant discrepancy between current guidelines regarding risk stratification. Despite the presence of a large aneurysm, our patient’s sudden cardiac death (SCD) risk was calculated as only 0.68% (low risk) according to the ESC 2014 risk score. However, the 2020 AHA/ACC guidelines correctly categorize this patient as high-risk (class IIa indication for an implantable cardioverter-defibrillator) based on the presence of an apical aneurysm alone. Our report provides concrete evidence of how morphological-only calculators can remain "blind" to life-threatening risks in this specific patient subset. We believe that this case will be of great interest to the readership, emphasizing both the technical nuances of echocardiographic assessment and the vital importance of guideline-directed risk management.

## Introduction

Although left ventricular apical aneurysms most commonly occur following acute myocardial infarction, hypertrophic cardiomyopathy (HCM) plays an important role in the etiology of non-ischemic aneurysms [1,2]. The presence of an aneurysmal structure is a significant risk factor in itself for intracavitary thrombus development, systemic embolic events, and life-threatening ventricular arrhythmias [1,2]. In the presented case, while increased wall stress and intraventricular pressure in apical HCM lead to chronic structural weakening of the apical myocardium, it is thought that the dynamic compression caused by the myocardial bridge in the distal segment of the LAD during the systolic phase may have further impaired apical myocardial perfusion.

## Case presentation

A 70-year-old male patient followed for apical HCM presented to our clinic with complaints of palpitations and dyspnea. On physical examination, his vital signs showed a blood pressure of 140/87 mmHg and a heart rate of 166 beats/min. The 12-lead ECG showed atrial fibrillation, left axis deviation, and deep T-wave inversions in the precordial leads (V4-V6). The patient's history included hypertension and non-critical peripheral artery disease, and an angiography performed within the last five years revealed plaques in the mid-segment of the left anterior descending artery (LAD) and a myocardial bridge causing 30-40% systolic narrowing in the distal segment.

**Figure 1:**
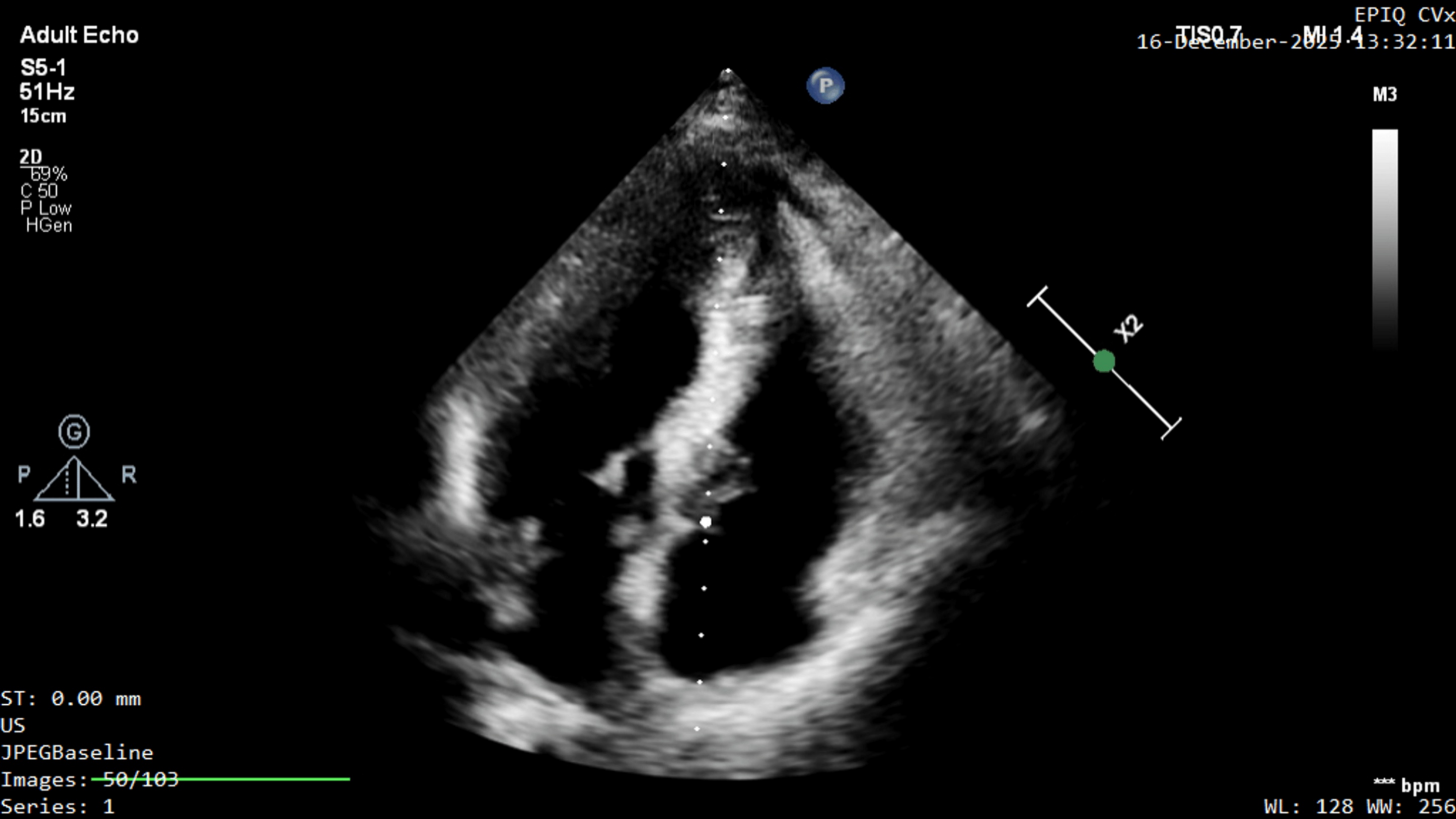
Standard apical four-chamber view in a patient followed for apical HCM, demonstrating marked apical thickening (2.0 cm). Note that the apical aneurysm is not visible in this standard imaging plane.

Transthoracic echocardiographic (TTE) examination was performed using an S5-1 probe and the Philips EPIQ CVx ultrasound system. Left ventricular ejection fraction (LVEF) was found to be 70%. On the apical four-chamber view, the left ventricular apical wall thickness was measured as 2.0 cm (Figure 1, Video 1).

**Video 1:** Transthoracic echocardiogram in the apical four-chamber view demonstrating marked apical hypertrophy with a maximal wall thickness of 2.0 cm. Notably, the apical aneurysm is not visible in this standard imaging plane.

**Figure 2:**
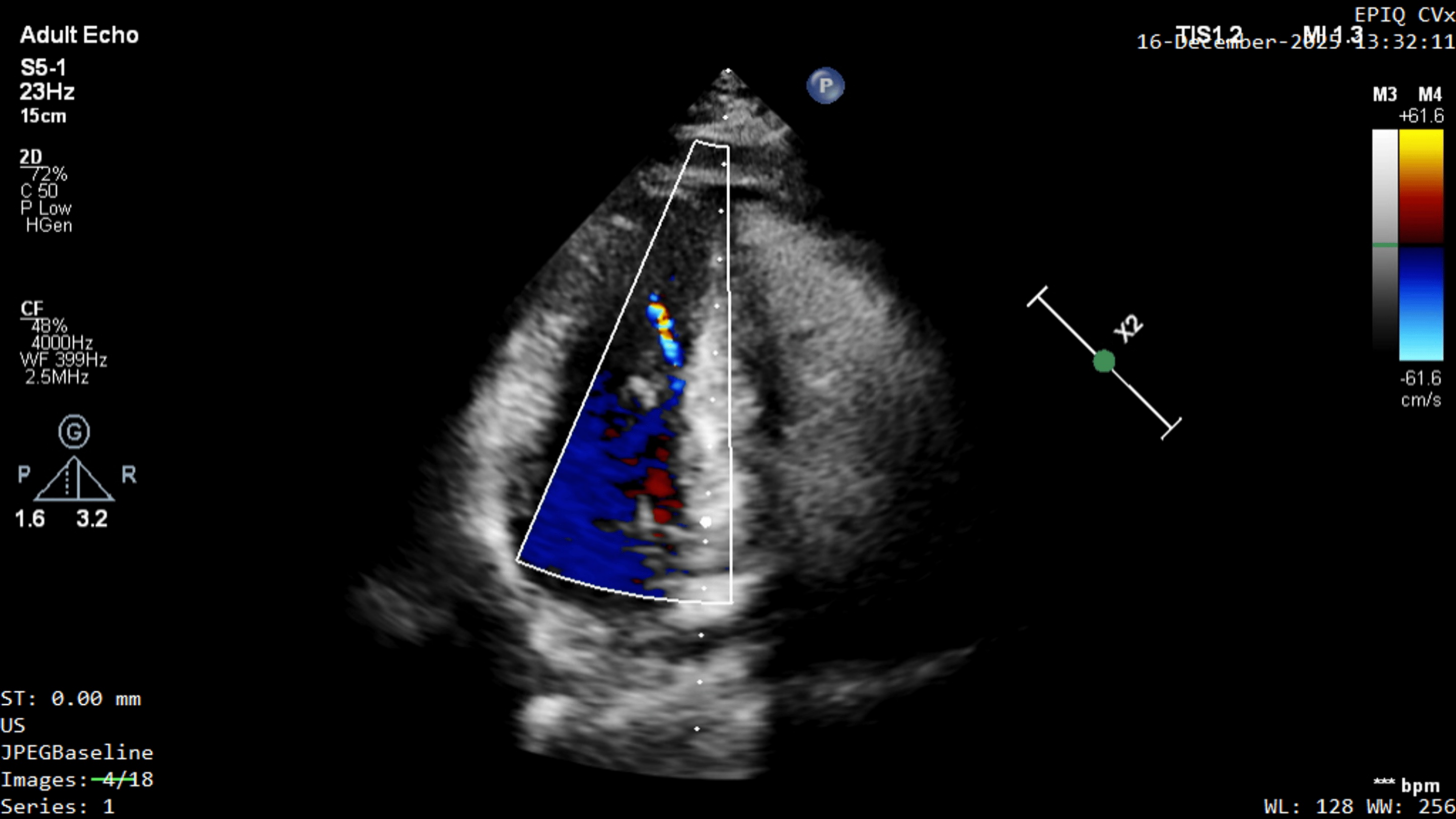
Identification of an apical jet suggesting a hidden aneurysm. Transthoracic echocardiography shows a modified apical four-chamber view with posterior angulation. Color Doppler imaging reveals an abnormal, high-velocity systolic jet originating from the left ventricular apex. The apical myocardial wall is markedly thickened (2.0 cm), raising clinical suspicion of an apical aneurysm, despite the absence of a visible aneurysmal sac in the standard four-chamber view.

A color Doppler jet was observed at the apex in a modified apical four-chamber view with posterior angulation, raising suspicion of an apical aneurysm (Figure 2, Video 2).

**Video 2:** Color Doppler echocardiography in a modified apical four-chamber view with posterior angulation. An abnormal systolic jet is visualized originating from the left ventricular apex, raising clinical suspicion for an underlying apical aneurysm.

**Figure 3:**
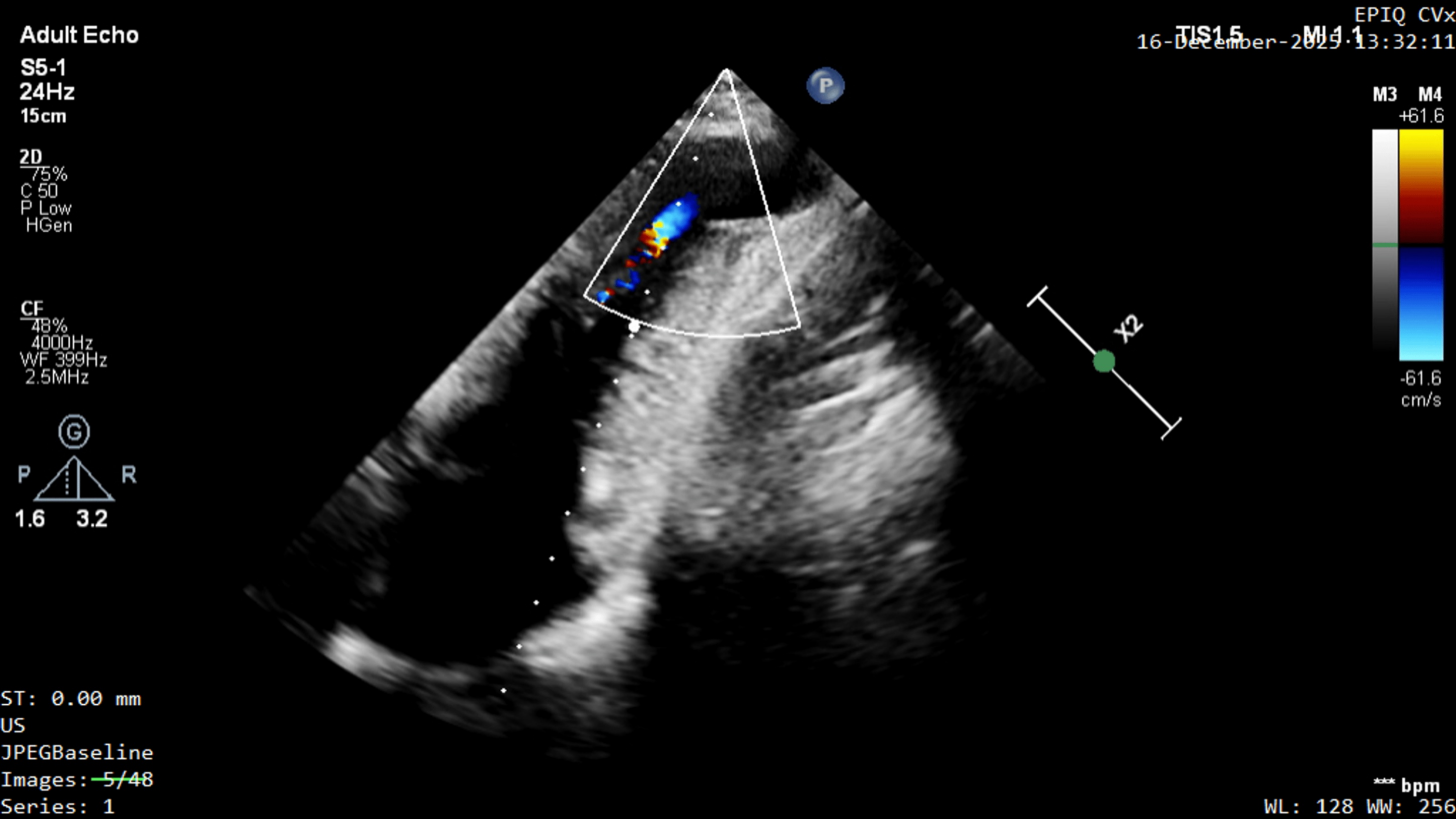
Following the absence of a visible aneurysm in the standard four-chamber view, detailed evaluation was performed in the apical two-chamber view. Meticulous intercostal "sliding" and long-axis "tilt" probe maneuvers successfully revealed an apical aneurysm connected to the previously noted color Doppler jet.

Since no aneurysm was visible in the apical four-chamber view, a detailed assessment was performed in apical two-chamber views; intercostal "sliding" of the probe and "tilt" along the long axis were performed, revealing an apical aneurysm connected to the jet (Figure 3; Video 3).

**Video 3:** Color Doppler confirmation of the aneurysmal jet. Dynamic color Doppler echocardiography in the apical two-chamber view clearly demonstrates the previously identified systolic jet communicating directly with the apical aneurysmal sac, confirming the diagnosis.

**Figure 4:**
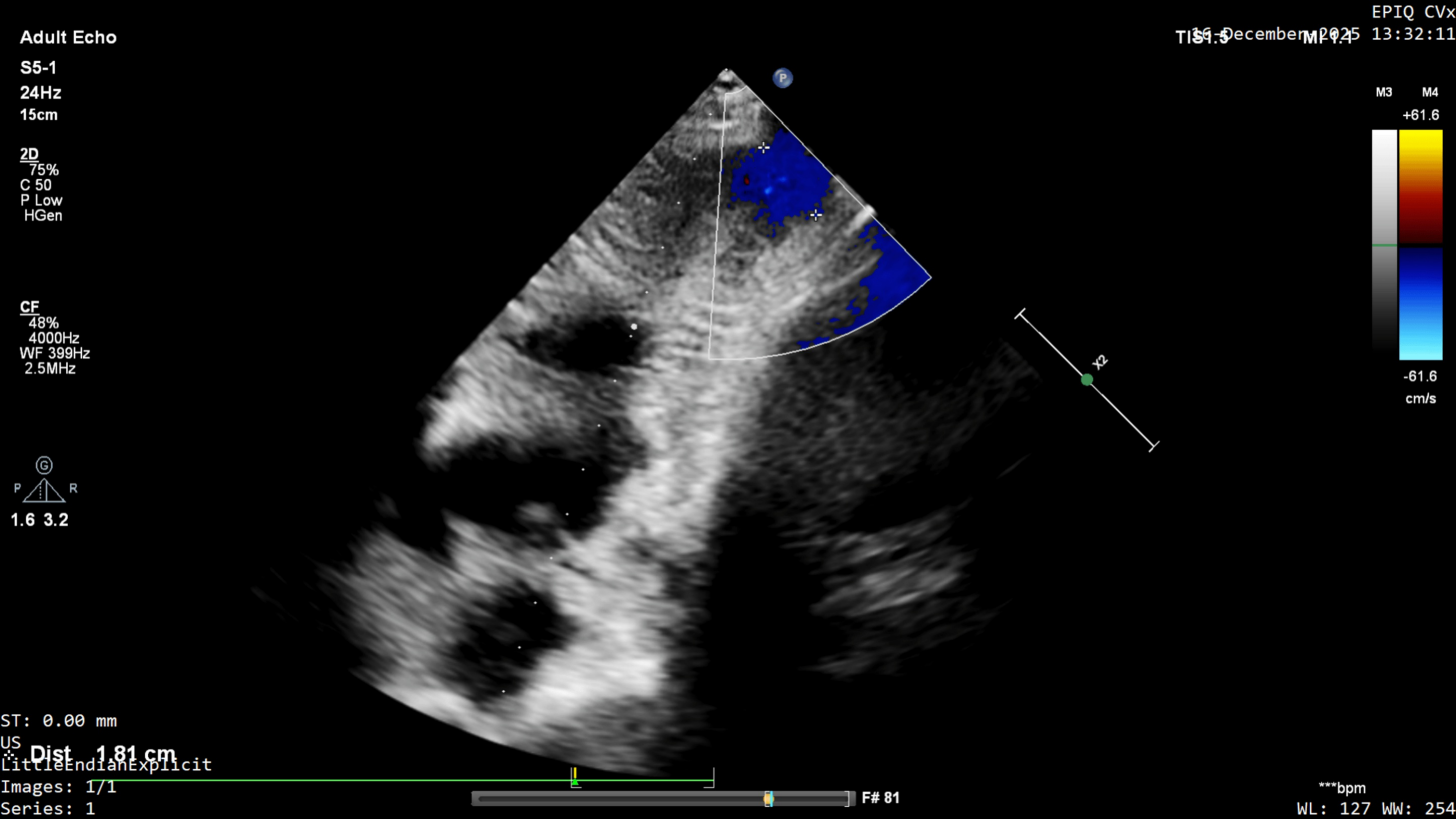
"Hourglass" appearance and aneurysm measurement. The left ventricular cavity demonstrates a characteristic "hourglass" appearance caused by the mid-apical hypertrophy and the distal aneurysmal sac. The apical aneurysm dimensions were measured as 1.9 x 1.8 cm.

The ventricular cavity took on a typical "hourglass" appearance due to the hypertrophic mid-apical segment and the distal aneurysm; the aneurysm diameter was measured as 1.9x1.8 cm (Figure 4).

The patient was followed up under anticoagulant therapy and arrhythmia control due to the high thromboembolic risk associated with the aneurysm. Regarding the risk of sudden cardiac death (SCD), the patient's HCM Risk-SCD score was calculated as 0.68% (low risk) [3]. However, the 2020 AHA/ACC (American Heart Association/American College of Cardiology) guidelines recognize the presence of a "left ventricular apical aneurysm" as a standalone major risk factor for sudden death, independent of mathematical risk scores [4]. Therefore, our patient had indication for an implantable cardioverter-defibrillator according to the 2020 AHA/ACC guidelines [4].

## Discussion

It has also been reported in the literature that the coexistence of myocardial bridge and HCM may facilitate aneurysm formation [5,6]. In our patient, severe apical hypertrophy and the sustained cavity obliteration inherent to the HCM phenotype are the primary drivers of structural weakening. However, we consider the myocardial bridge a significant aggravating factor; the dynamic compression it causes during systole likely further impairs the precarious apical perfusion. Rowin et al. demonstrated that the risk of SCD and thromboembolism is increased in HCM patients with apical aneurysms [2]. In our case, although the LVEF was 70%, the deceleration of blood flow within the aneurysmal sac poses a risk of thrombus [7]. Current literature emphasizes the importance of prophylactic anticoagulation and arrhythmia monitoring in these patients [4].

Although echocardiography is the first diagnostic step, it has been shown that the detection rate of apical aneurysms is significantly higher when cardiac magnetic resonance (CMR) is used [8]. Furthermore, the fibrotic transition zone around the aneurysm demonstrated by CMR creates an ideal focus for "re-entry" arrhythmias [2]. When examining the difference between guidelines, the fact that the AHA guidelines recognize apical aneurysm as a standalone major risk factor is concrete evidence that the European Society of Cardiology (ESC) calculator, which relies solely on morphological measurements, is inadequate for patients with apical aneurysms [4,9]. Unfortunately, CMR imaging could not be performed in our patient due to persistent atrial fibrillation, which severely degrades CMR image quality through ECG-gating artifacts. This remains a limitation of our case. However, the inability to perform the gold standard CMR makes the successful detection and measurement (1.9x1.8 cm) of the aneurysm solely via persistent TTE maneuvers even more clinically significant.

## Conclusions

Apical HCM is an insidious condition that manifests with deep T-wave inversions on precordial ECG, yet it can often be overlooked. In the presence of any sign, such as an apical jet, an aneurysm should be searched for in different windows and by modifying the probe. Even if traditional risk scores are low, guideline-directed (AHA) risk management, prophylactic anticoagulation, and rhythm monitoring are of vital importance in these patients.
